# Congratulations to the 2026 Abstract Award Winners

**DOI:** 10.1002/pmf2.70190

**Published:** 2026-02-09

**Bruce A. Work Award for Best Research by a Practicing or Training Maternal‐Fetal Medicine Physician outside of the US**

32: Antenatal Risk Factors for Neonatal Hypoxic‐Ischaemic Encephalopathy: A Prospective National Population‐Based Case‐Control Study

**Pierre Delorme, MD, PhD**

**Disparities Award for Best Research on Diversity/Disparity in Health Outcomes**

45: Impact of a substance use screening program on racial disparities in urine toxicology testing

**Mariam Naqvi, MD**

**Dru Carlson Memorial Award for Best Research in Ultrasound and Genetics**

70: Unsupervised Machine Learning of Fetal Myocardial Performance Index Trajectories Identifies FGR and SGA Patterns

**Rebecca Horgan, MD**

**Norman F. Gant Award for Best Research in Maternal Medicine**

1: A Randomized Controlled Trial of Baby2Home: A Postpartum Digital Mental Health Intervention

**Emily S. Miller, MD, MPH**

**The SMFM Outstanding Early Career Investigator Travel Award supported by Dr. Laxmi Baxi**

40: Intraplacental Gene Transfer of IGF‐1 Corrects Preeclampsia in BPH/5 Mouse

**Kristen L. Moriarty, MD**

**Scholarship for Research in TAPS/Monochorionic Twins supported by the Kaiden Paul Foundation**

33: Fetal hematologic profile in monochorionic twins with anemia‐polycythemia sequence undergoing serial transfusion and partial exchange

**Alexandra D. Forrest, MD**

